# The role of fibromodulin in cancer pathogenesis: implications for diagnosis and therapy

**DOI:** 10.1186/s12935-019-0870-6

**Published:** 2019-06-10

**Authors:** Mohammad Hossein Pourhanifeh, Rezvan Mohammadi, Somaye Noruzi, Seyede Atefe Hosseini, Sahar Fanoudi, Yousef Mohamadi, Milad Hashemzehi, Zatollah Asemi, Hamid Reza Mirzaei, Reza Salarinia, Hamed Mirzaei

**Affiliations:** 1 Research Center for Biochemistry and Nutrition in Metabolic Diseases, Kashan University of Medical Sciences, Kashan, Iran; 2 Department of Medical Biotechnology and Molecular Sciences, School of Medicine, North Khorasan University of Medical Sciences, Bojnurd, Iran; 3 Department of Pharmacology, Faculty of Medicine, Mashhad University of Medical Sciences, Mashhad, Iran; 4 Department of Anatomy, Faculty of Medicine, Qom University of Medical Sciences, Qom, Iran; 5 Iranshahr University of Medical Sciences, Iranshahr, Iran; 6 Division of Neurocognitive Sciences, Psychiatry and Behavioral Sciences Research Center, Mashhad University of Medical Sciences, Mashhad, Iran; 7 Department of Medical Immunology, School of Medicine, Tehran University of Medical Sciences, Tehran, Iran

**Keywords:** Fibromodulin, Diagnosis, Therapy, Cancer

## Abstract

Fibromodulin (FMOD) is known as one of very important extracellular matrix small leucine-rich proteoglycans. This small leucine-rich proteoglycan has critical roles in the extracellular matrix organization and necessary for repairing of tissue in many organs. Given that the major task of FMOD is the modulation of collagen fibrillogenesis. However, recently observed that FMOD plays very important roles in the modulation of a variety of pivotal biological processes including angiogenesis, regulation of TGF-β activity, and differentiation of human fibroblasts into pluripotent cells, inflammatory mechanisms, apoptosis and metastatic related phenotypes. Besides these roles, FMOD has been considered as a new tumor-related antigen in some malignancies such as lymphoma, leukemia, and leiomyoma. Taken together, these findings proposed that FMOD could be introduced as diagnostic and therapeutic biomarkers in treatment of various cancers. Herein, for first time, we highlighted the various roles of FMOD in the cancerous conditions. Moreover, we summarized the diagnostic and therapeutic applications of FMOD in cancer therapy.

## Introduction

Cancer is caused by uncontrolled division of abnormal cells which are able to invade other tissues. It is known as a result of an imbalance between cell proliferation and cell death rate caused by mutations in the regions of DNA which code regulator proteins [1]. None isolated cells could survive and all cell types need to reside in extracellular matrix (ECM) and interact with ECM components. The ECM components play important roles in tumor formation, as well. Cancer development and its progression have been associated with an increase in the ECM deposition. The ECM serves not only as a 3-D scaffold required for tissue organization, but also induces a series of chemical and physical signals which are crucial for survival, proliferation and differentiation of cancer cells as well as vascular development and invasion of tumor cells [2]. For instance, the ECM is a reservoir for various angiogenic growth factors and proteases. ECM transduces these signals via ECs integrins and regulates their angiogenic phenotype [3]. Stromal cells have been recognized as a major source of ECM proteins. However, more recently, some studies introduced the cancer cells as active and important components involved in ECM remodeling [4].

Proteoglycans are relatively small components of ECM belonging to the small-leucine-rich-proteoglycan (SLRP) family [5], which are categorized into classes I–V [6]. Several proteoglycans, such as decorin (Dcn) and biglycan from class I, and fibromodulin (FMOD) and lumican from class II, contribute to the regulation of collagen fibrillogenesis [7]. Among these proteoglycans, FMOD has been emerged with attractive features which lead to affecting on a wide ranges of critical biological processes such as angiogenesis, apoptosis, and migration. Multiple lines evidence indicated that FMOD exerts its effects via targeting certain signaling pathways such as TGF-β [8]. This proteoglycan could play very important roles in the initiation and progression of several malignancies [9]. Hence, FMOD could be employed as diagnostic and therapeutic biomarkers in treatment of different cancers. In the current review, for first time, we highlighted the various roles of FMOD in the cancerous conditions. Moreover, we summarized the diagnostic and therapeutic applications of FMOD in cancer therapy.

## FMOD and cancer

FMOD is a main proteoglycan which contributes to remodeling of the ECM through binding to matrix molecules, thereby plays an essential role in tissue repair, tumor progression and cancer [10]. The interaction between this proteoglycan and lysyl oxidase (LOX; collagen cross-linking enzyme) regulates the ECM composition leading to supply an appropriate environment for cellular turnover [11]. Moreover, FMOD involves in vasculature development and regeneration [12], tumor growth suppression and apoptosis prevention [13]. It has also been recognized as a growth factor which modulates there programming of somatic cells toward the pluripotent state [14].

**Fig. 1:**
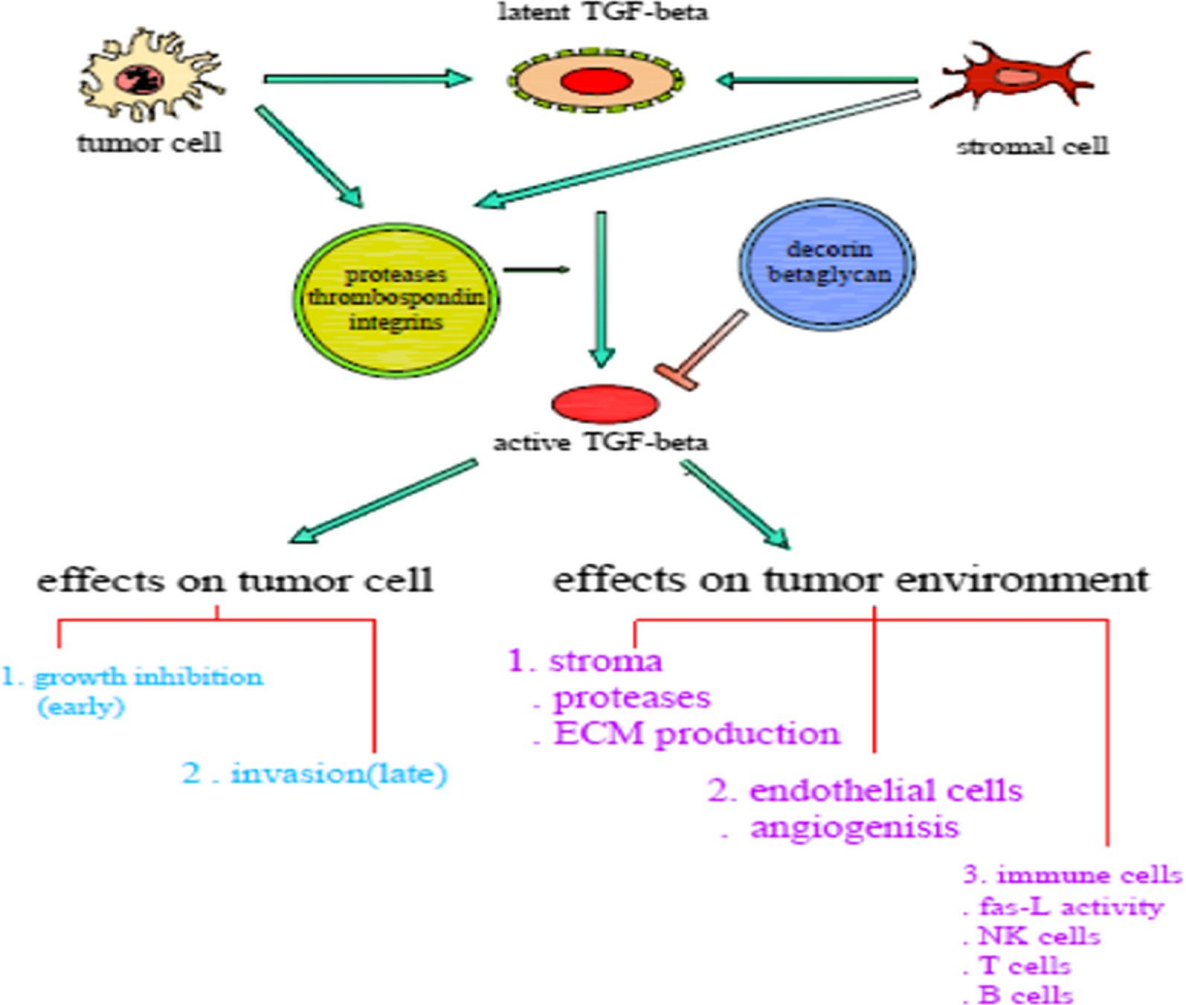
TGF-β signaling in cancer progression and tumor suppression

It is believed that FMOD interaction with transforming growth factor (TGF)-β, a key profibrotic cytokine, keeps this growth factor within the ECM to regulate the action of local TGF-β (Fig. 1). Due to its prominent role in regulation of cell growth, differentiation and migration, TGF-β is well-known as a key contributor in cancer progression. It is a double-edged sword which mediates both tumor suppression and promotion through instigating the cellular phenotypical changes [15]. Even though this growth factor is known to inhibit cell proliferation and induce apoptotic cell death during the early stages of tumorigenesis, tumor cells may lose their growth-inhibitory response to TGF-β resulting in inducing epithelial-to-mesenchymal transition and promoting cell migration [3, 15].

**Fig. 2:**
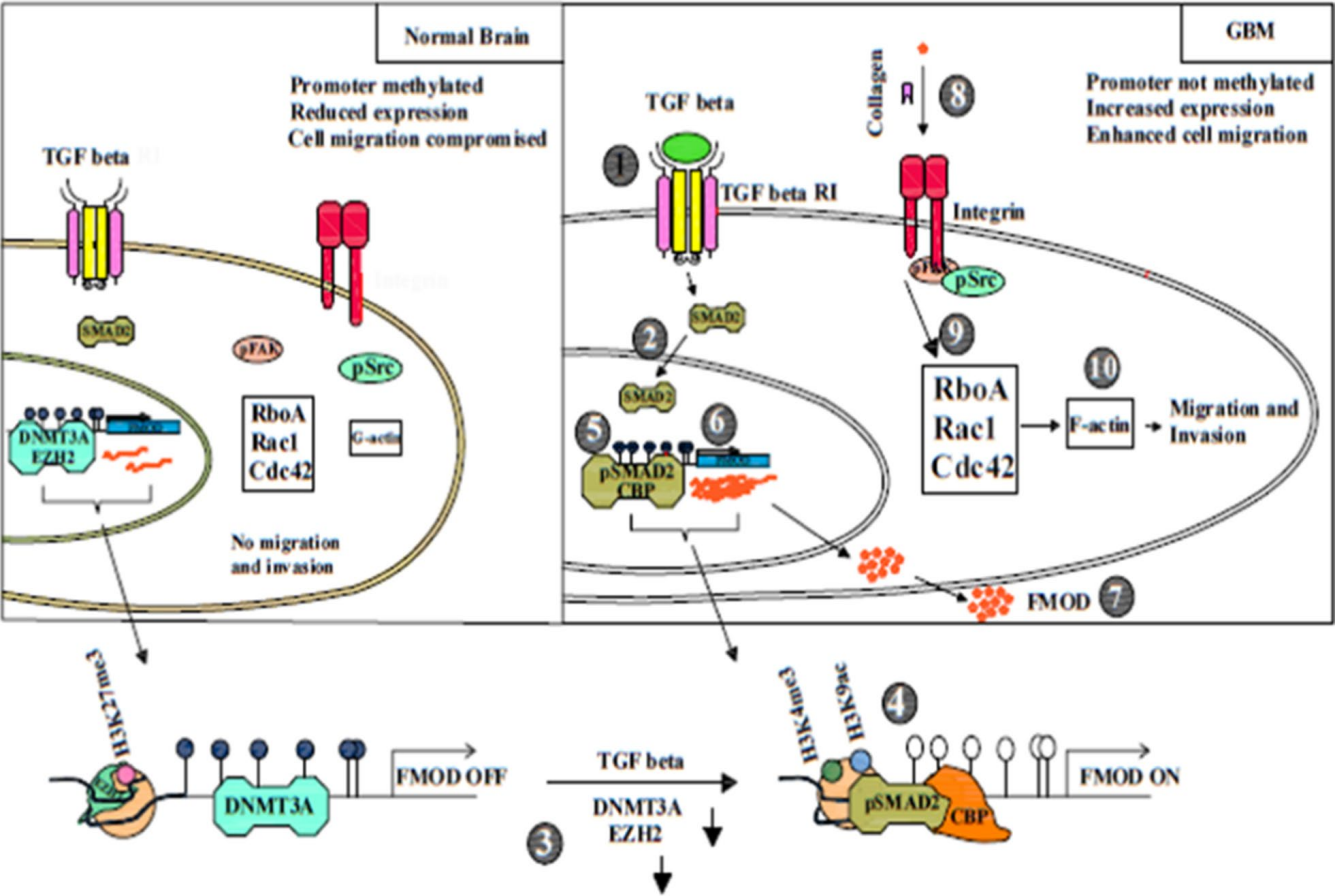
Fibromodulin as a pivotal gene for glioma cell migration

In addition to its effects on the tumor cells behavior, TGF-β inhibits cell adhesion to the ECM, promotes extracellular matrix degradation, and induces immune suppression as well as angiogenesis. Through these mechanisms, TGF-β modulates the metastasis process [3]. Increased secretion of protease and plasmin from tumor cells enhances the activation of TGF-β which causes extracellular matrix degradation with a subsequent release of stored TGF-β. This increased activation of TGF-β profoundly affects the micro-environment of tumor tissue [16]. TGF-β recruits and activates some intracellular signaling pathways including the expression of several genes, such as fibronectin and collagen, specifically Smad and mitogen-activated protein kinase (MAPK)/extracellular signal-regulated kinase (ERK) pathways. Additionally, FMOD expression is differentially regulated by TGF-β [17]. FMOD function, as a regulator of TGF-β, has been observed in scarless wound healing and collagen assembly in skin development [18, 19]. In addition, FMOD shows potential antagonist effects on TGF-β regulation, compared with Dcn, in the inhibition of neointimal hyperplasia in saphenous vein graft [20]. It is also implicated in the inhibition of nuclear factor-κB (NF-κB) signaling (a family of transcription factors) through suppressing the IκBα protein [21]. Dissociation of IκB domain due to NF-κB activation allows the NF-κB dimers to change gene expression and locate in the nucleus [22]. Additionally, NF-κB signaling is essential for epithelial–mesenchymal transition (EMT) and its therapeutic inhibition may mitigate tumor invasion and metastasis [21]. Besides different pre-clinical studies on FMOD, many clinical studies have highlighted the role of FMOD in cancer pathogenesis and its diagnostic roles (Fig. 2).

In a study, Mayr et al., indicated that there were a significant up regulation of FMOD in chronic lymphocytic leukemia (CLL) cells than normal B lymphocytes [23]. Authors revealed that T cells number were elevated 2- to 3.5-fold and the number of T cells recognizing FMOD peptides bound to HLA-A2 dimers were elevated 10-fold during 4 weeks in vitro. Taken together, these findings proposed that FMOD might be known as potential tumor-associated antigen (TAA) in CLL which is able to affect on FMOD-specific T cells expansion [23].

Given that FMOD and other members of the proteoglycan family could be involved in collagen fibrillogenesis and cell adhesion and they also help to suppression of tumor growth, regulation of cytokine activity, and prevention of apoptosis [24]. FMOD has been known as one of TAA in B-CLL which is able to provide a specific anti-tumor response. Hence, FMOD is potential candidate for cancer immunotherapy. In a study, Hassan et al., assessed FMOD expression in 30 B-CLL subjects and showed the pathogenic roles of FMOD in these patients [24]. Their results indicated that there was a significant expression of FMOD in B-CLL subjects than control group. FMOD was expressed in 46.7% of B-CLL subjects. Moreover, authors showed that there were a significant relationship between up regulation of FMOD and some risk factors which were studied on B-CLL subjects (i.e., lower haemoglobin level, lower platelet count, hepatomegaly, and lower RBCs count) and borderline showed a significant relationships with other risk factors such as splenomegaly and lymphadenopathy. These findings suggested that FMOD has critical roles in the pathophysiology of CLL [24].

### FMOD induces and suppresses apoptosis

The critical roles of FMOD in the pathogenesis of cancer had been ignored for a long time. Now, it is clear that the regulation of collagen fibrillogenesis and cell adhesion are not the only roles of FMOD and the other proteoglycans, but they also contribute to apoptosis prevention [30, 40]. It has been shown that FMOD modulates and suppresses the functions of TGF-β, both in vivo and in vitro. For example, it seems that TGF-β binding to its binding sites on FMOD inhibits the apoptotic activity of TGF-β in the B-cell chronic lymphocytic leukemia (B-CLL) [30].

NF-κB signaling is another pathway involved in apoptosis because its constitutive activation induces cell proliferation and inhibits apoptosis [41]. Under normal condition, the NF-κB signaling pathway is inhibited by a group of inhibitory molecules termed IκB proteins. These proteins, such as IκBα, attach to NF-κB to suppress its activation in the cytoplasm [42]. FMOD suppresses the NF-κB signaling through delaying the degradation of IκBα protein byc-Jun N-terminal kinase activation, suppression of calpain and casein kinase 2 activity, and apoptosis induction [21]. Available data suggests that the constitutive turnover of IκBα is mediated by CK2-calpain signaling axis which is inactivated in FMOD-expressing fibroblasts in a JNK-dependent manner. FMOD expression is essential for JNK-dependent caspase-3 and caspase-7 activation in 3T3-L1 fibroblasts, suggesting that FMOD-mediated activation of JNK promotes apoptosis in fibroblasts [41].

### FMOD as a promoter for angiogenesis

FMOD is an effective angiogenic factor with important roles in cell fate determination. FMOD plays a key role in angiogenesis in lung cancer, wound healing, optical and cutaneous diseases related to angiogenesis [8, 16, 43]. It increases the expression of angiogenic growth factors angiopoietin 2 (ANG2) and VEGF, and inhibits the expression of vascular stabilizing factor ANG1. These effects indicate that FMOD may regulate further signaling cascades related to growth factors than just TGF-β pathway [44].

These angiogenic regulatory effects strongly suggest that autocrine-secreted FMOD by cancer cells has a stimulating role in tumor angiogenesis of small-cell lung cancer. It facilitates the angiogenic phenotype transformation of endothelial cells as a pro-angiogenic step [8]. The role of FOMD in angiogenesis suggests its potential abilities in cancer therapy, wound healing, and other conditions associated with abnormal angiogenesis [12, 45].

NF-κB modulates the transcription of several target genes implicated in invasion angiogenesis, apoptosis, and migration [46, 47]. It has been established to possess important roles in the progression of breast cancer, cell proliferation control and oncogenesis [48]. Glial Cell Line-Derived Neurotrophic Factor **(**GDNF) is a member of the TGF-β family. GDNF induces the expression of VEGF-C in neu-roblastoma cells and promotes angiogenesis in renal cells, normal skin cells, and hepatocellular carcinoma cells [49–52]. Chen et al. [53] conducted an in vitro study to assess the molecular mechanisms of FMOD expression induced by GDNF. To reveal the role of GDNF in FMOD expression, they silenced the expression of GDNF in U251 GBM cells. It was showed that GDNF expression silencing suppressed the protein and mRNA expression of FMOD. They also demonstrated that the secretion of VEGF, as an important pro-angiogenic factor, transfected with si-FMOD was significantly decreased in U251 cells. Furthermore, they indicate that GDNF contributes to FMOD promoter demethylation. Totally, GDNF promotes FMOD expression in GBM cells.

Therefore, the main regulatory roles of FMOD in angiogenesis and apoptosis are associated with TGF-β superfamily as well as NF-κB signaling pathway. Consequently, new therapeutic medications could be produced to inhibit the development and progression of malignancies through regulating these pathways and direct interaction with mentioned agents.

### FMOD promotes cell migration

FMOD is regulated epigenetically through the methylation of its promoter. It induces the formation of filamentous actin stress fiber leading to promote the migration of glioma cells [31].

The integrins have a main role in linking intracellular actin cytoskeleton to the extracellular matrix throughout cell migration process [54, 55]. The interaction of integrin with extracellular matrix is transduced into cells by activation of integrin-related proteins. Downstream to integrins, FAK-Src, as a non-receptor tyrosine kinase, has a crucial role in signaling to Rho GTPases [55]. Rho GTPases family, including Rho, Rac and Cdc42, contributes to cytoskeleton remodeling, cell motility and cell migration [56]. It has been recently observed that FMOD silencing significantly inhibits the TGF-β1-mediated migration of glioma cells (Fig. 1) [31].

Mondal and colleagues demonstrated that tumor secreted FMOD activates integrin-FAK-Src-Rho GTPase-dependent signaling to induce the migration of glioma cells. They also observed that the remodeling of actin cytoskeleton is critical for glioma cell migration induced by FMOD [31].

## FMOD as a potential novel biomarker

Since effective treatments of early stages of diseases are available, new molecular biomarkers are urgently needed for an opportune diagnosis and adequate prediction of the clinical course of disease [57]. The FMOD gene has been considered as a potential biomarker to be more evaluated in clinical samples from patients diagnosed with benign or malignant prostatic cancers. Validation of FMOD transcript in a large population is required to ascertain its usefulness as a biomarker of cancer. FMOD encodes a proteoglycan which can be detected in different body fluids, such as blood, urine or prostatic secretions. Therefore, due to FMOD expression in protein level, it is able to differentiate prostate cancer patients from the patients with benign disease [34]. Moreover, it has been demonstrated that FMOD gene is up-regulated in highly malignant glioblastoma compared with relatively benign pilocytic astrocytoma [32]. According to this correlation, FMOD could be utilized as a biomarker, which indicates the disease severity, at least in the brain tumors [44]. It seems that more studies could contribute to introduce FMOD as potential biomarker to clinical setting in near future.

## Targeting FMOD as therapeutic approach in cancer

FMOD has been recognized as a novel tumor-associated antigen in lymphoma, leiomyoma, and leukemia [17, 23]. FMOD mRNA has been detected different clinical malignancies, such as breast, prostate, and lung carcinomas [58–60]. FMOD deficiency decreases interstitial fluid pressure and enhances extracellular volume in experimental carcinomas and results in declined collagen fibril thickness in experimental carcinoma. It has been previously indicated that FMOD regulates collagen fibers in extracellular environment of tumor [37]. In various cancers, the altered small leucine-rich proteoglycans (SLRPs) expression, such as decorin, biglycan, and FMOD, has been suggested as a diagnostic, prognostic and therapeutic tool (Tables 1, 2) [61]. Recently, it has been shown that FMOD, along with other SLRP family members, is not only implicated in cell adhesion and collagen fibrillogenesis, but also contributes to regulate the tumor growth suppression, transcription factors activity, and apoptosis prevention [42, 61]. FMOD transcript over expression was described in B-cell chronic lymphocytic leukemia (CLL-B), and it has been suggested as a target for therapeutic approaches in this cancer [23, 24, 30].

**Table 1:** Role of FMOD in various cancer

Cancer type	Model	Expression	Function/signaling pathway	Ref
CLL	Human	Up regulation	Expansion of specific CD8 autologous T lymphocytes	[23]
	Human	Up regulation	Collagen fibrillogenesis and cell adhesion and contribute to modulation of cytokine activity, suppression of tumor growth, and prevention of apoptosis	[24]
	Human	Up regulation	Binds TGF-β and typically negatively modulates its activity overexpression in CLL	[25]
	Human	Up regulation	modulation of TGF-β signaling and cell adhesion	[26]
	Human	Up regulation	Among the four patients with increase of CTL was observed after the fourth and sixth dendritic cell vaccination	[27]
	Human	Up regulation	Tumor progression	[28]
	Human	Up regulation	Tumor progression	[29]
	Human	Up regulation	Activation of normal B and T lymphocytes, tonsil B cells, CLL B cells, and B-CLL cell lines	[30]
GBM	Cell line	Up regulation	Induce migration, promoter methylation and transcript, actin cytoskeleton remodeling/FAK-Src-Rho-ROCK signaling	[31]
	Human	Up regulation	Generation of neoplastic ECM and induce tumor progression and invasion	[32]
	Human	Up regulation	The methylation of FMOD promoter is correlated with good prognosis	[33]
Prostate cancer	Cell line	Up regulation	Modulate the activity of transcription factors, suppression of tumor growth and apoptosis prevention	[34]
	Human	Up regulation	Regulation of angiogenesis, reprogramming of human fibroblasts into pluripotent cells, modulation of TGF-β activity, inflammatory processes and association with metastatic phenotypes	[9]
Colon cancer	Mouse	Down regulation	modulation of collagen fibrils in tumor stroma	[35]
	Cell line	Up regulation	Increasing of ECM density that inhibit the migration and invasion/collagen types I, III, IV and V, biglycan	[36]
	Mice	Up regulation	Prompt the formation of a dense stroma and an elevated interstitial fluid pressure	[37]
Leiomyoma	Human	Up regulation	Connective tissue remodeling, specifically fibrillogenesis, cell–cell adhesion and modulation of cytokine autocrine/paracrine actions	[17]
	Human tumor	Up regulation	transfection of the cells with Smad3 SiRNA resulted in significant reduction in TGF-β-induced FMOD TGF-β, through Smad and MAPK signalling pathways, regulated the expression of FMOD	[17]
Myometrium	Human	Up regulation	FMOD regulated by gonadotropin-releasing hormone analogue therapy and TGF-β through Smad and MAPK-mediated signaling. Connective tissue remodeling, specifically fibrillogenesis, cell–cell adhesion and modulation of cytokine autocrine/paracrine actions	[17]
SCLC	Cell line	Down regulation	Inhibit proliferation, migration, and invasion, angiogenesis/VEGF, TGF-β1, FGF-2, and PDGF-B	[38]
MCL	Human	Up regulation	Activation of normal B and T lymphocytes, tonsil B cells	[30]
Myxoma	Human	Up regulation	Diagnostic usefulness	[39]
Insulinoma	Mice	Up regulation	Inhibition of expression by anti-inflammatory agents showed that FMOD promoted the formation of a dense stroma and an elevated interstitial fluid pressure	[37]
Thyroid cancer	Mice	Up regulation	Inhibition of expression by anti-inflammatory agents showed that FMOD promoted the formation of a dense stroma and an elevated interstitial fluid pressure	[37]

*CLL* chronic lymphocytic leukemia, *MCL* mantle cell lymphoma, *SCLC* small cell lung cancer, *GBM* glioblastoma, *ECM* extracellular matrix

**Table 2:** FMOD gene therapy in cancer

Cancer type	Model	Strategy	Function(s)	Citation
CLL	Cell line	Using siRNA against FMOD	Induce apoptosis, cells aggregated together and appeared to be granular	[40]
Breast cancer	Cell line	Using recombinant Adenovirus FMOD	Suppresses NF-κB DNA binding and TGF-β1 that control of cell proliferation and oncogenesis	[21]
Leukemia	Cell line	Using siRNA against FMOD	Diminished the apoptosis of B-CLL cells	[62]
Glioblastoma	Cell line	Using RNAi against FMOD	Induce glioma cell migration and invasion by promoting actin cytoskeleton remodeling pathway	[31]

In a study, Choudhury et al., assessed the inhibition of FMOD and ROR1 in CLL cells [40]. Given that FMOD and ROR1 are two genes which are over expressed in CLL cells than normal blood B cells. They used siRNAs to specifically inhibit expression of FMOD and ROR1 in human fibroblast cell lines, healthy B cells and CLL cells Their results indicated that utilization of siRNA is able to induce a specific decreasing (75–95%) in ROR1 and FMOD expression at mRNA levels. Further analysis indicated that 48 h after siRNA treatment the FMOD and ROR1 were significantly down regulated at protein levels. Moreover, suppress expression of ROR1 and FMOD by using specific siRNAs could be associated with significant decreasing of apoptosis of CLL cells but not of B cells which was isolated from normal subjects. On the other hand, when human fibroblast cell lines were treated by ROR1 and FMOD siRNA, there were no observed apoptosis. These findings suggested that FMOD and ROR1 could be associated with the survival of CLL cells and these could be introduced as therapeutic targets in the treatment of CLL [40]. In an in vitro study conducted by Dawoody Nejad et al. [21], it was established that FMOD over expression is related to TGF-β1 and NF-κB down-regulation in metastatic breast cancer cells.

In recently study, Mondal and colleagues indicated that FMOD as a GBM over expressed gene because of the loss of promoter methylation [31]. They showed that the released FMOD enables to induce glioma cell migration via its ability to enhance formation of filamentous actin stress fiber. Utilization of cytochalasin D which is an inhibitor for actin polymerization, could significantly decrease the FMOD-induced glioma cell migration. In addition, using siRNA and inhibitor-based small molecules revealed that integrin-FAK-Src-Rho-ROCK signaling pathway is very important signaling pathway which is related to FMOD-induced glioma cell migration. It has been showed that FMOD without C-terminus LRR11 domain (ΔFMOD) is not able to bind collagen type I and could not also induce integrin and glioma cell migration. A 9-mer wild-type peptide originated from the FMOD C-terminus could inhibit the activation of FMOD-induced integrin and migration. The Chromatin immunoprecipitation-PCR analyses indicated that transforming TGF-β1 is able to modulate expression of FMOD via epigenetic FMOD promoter remodeling. The silencing of FMOD is associated with inhibition of TGF-β1-mediated GBM cell migration. Multivariate Cox regression analysis, demonstrated that promoter methylation and transcript levels of FMOD could predict prognosis in GBM. Collectively, using specific siRNA against FMOD could show therapeutic effects via inhibition of TGF-β1 pathway. Thus, FMOD is a potential target in treatment of GBM [31].

## Conclusion

Current data suggest the important role of FMOD in the pathogenesis of cancer. This proteoglycan interacts with cellular and molecular mechanisms, such as NF-kB, to develop malignancy. FMOD acts through increasing the migration and angiogenesis to progress cancer. It also suppresses the apoptosis induction.

Despite several attempts on this horizon, studies on FMOD are associated with some limitations. It seems that more pre-clinical experiments and clinical studies (with large samples) could contribute to more understanding of FMOD roles in the cancer pathogenesis. Moreover, more assessments could help to introduce this molecule as diagnostic or prognostic biomarkers for monitoring cancer subjects. This review suggest, evaluation of FMOD in all cancerous conditions may lead to introduce it to clinical settings.

## Data Availability

The primary data for this study is available from the authors on direct request.
